# Second stage of labor beyond 4 h in nulliparous patients with epidural analgesia: implications and outcomes—a retrospective cohort

**DOI:** 10.1007/s00404-025-08141-0

**Published:** 2025-08-05

**Authors:** Gal Cohen, Michal Kovo, Hila Shalev-Ram, Chen Key-Segal, Gil Shechter-Maor, Tal Biron-Shental, Hanoch Schreiber

**Affiliations:** 1 Department of Obstetrics and Gynecology, Meir Medical Center, 59 Tchernichovsky St., 44281 Kfar Saba, Israel; 2 School of Medicine, Faculty of Medical and Health Sciences, Tel Aviv University, Tel Aviv, Israel; 3 Shamir Medical Center (Assaf Harofeh), Be’er Ya’akov, Israel

**Keywords:** Prolonged second stage of labor, Failed vacuum extraction, Shoulder dystocia, Subgaleal hematoma, Nulliparous, Epidural anesthesia

## Abstract

**Purpose:**

To evaluate the obstetric outcomes in nulliparas with epidural who exceeded the 95th percentile duration of 4 h.

**Methods:**

This retrospective cohort included all term, singleton deliveries of nulliparas with epidural analgesia and second stage duration > 3 h, from 2014 to 2021. Maternal and neonatal outcomes were evaluated by comparing second stage duration 3–4 h vs. > 4 h.

**Results:**

A total of 2,798 deliveries were included, with 2273 in the 3–4 h group (mean duration 3.42 ± 0.28 h) and 525 in the > 4 h group (mean duration 4.38 ± 0.42 h). Compared to the 3–4 h group, the > 4 h group had lower rate of vaginal deliveries (80.4% vs. 93%, p < 0.001), relatively higher rate of vacuum extractions (VE) (56.0% vs. 42.4%, p < 0.001) and higher rate of cesarean deliveries (CD) (19.6% vs. 7.0%, p < 0.001). The > 4 h group had higher rates of macrosomia and large for gestational age birthweights (7.0% vs. 4.0%, p < 0.003 and 11.8% vs. 8.9%, p = 0.039, respectively). Shoulder dystocia, neonatal subgaleal hematoma and failed VE were more common in the > 4 h group (2.7% vs. 1.1%, 9.0% vs. 3.3% and 4.4% vs. 1.0%, respectively, p < 0.01 for all), as well as the composite neonatal trauma outcome (11.2% vs. 4.1%, p < 0.001). Multivariable logistic regression adjusted for confounders including obesity, hypertensive disorders, diabetes, macrosomia and delivery mode, revealed that a duration of > 4 h was associated with increased risks of shoulder dystocia, subgaleal hematoma and failed VE.

**Conclusion:**

Most patients achieve vaginal delivery even after a duration of > 4 h. However, it is associated with increased risks of neonatal birth trauma and failed VE**.**

## What does this study add to the clinical work

**Table:** 

This study provides robust evidence that prolonging the second stage of labor beyond 4 hours in nulliparas with epidural analgesia increases the risk of neonatal birth trauma and failed vacuum extraction, despite maintaining high vaginal delivery rates. These findings highlight the need for individualized decision-making and thorough patient counseling when considering second-stage extension.	

## Introduction

Cesarean delivery (CD) during the second stage of labor occurs in about 2–8% of all births and poses greater risks to both the mother and neonate compared to first-stage CDs [1, 2]. Complications include unintended extensions, intraoperative trauma, blood transfusions, longer procedure duration and hospital stay, maternal endometritis, lower Apgar scores, neonatal trauma, perinatal asphyxia, and death [3–7].

To reduce primary CD rates, the American College of Obstetricians and Gynecologists (ACOG) and the Society for Maternal–Fetal Medicine updated labor dystocia definitions in 2014 [8]. Incorporating Zhang’s labor curves [9], they allowed a prolonged second stage for nulliparas up to 3 h, longer with epidural analgesia. They argued that the risks of adverse neonatal outcomes from prolonged second-stage of labor appear low and incremental and acknowledged no maximum time limit for the second stage. Other societies have adopted these recommendations, allowing a second stage of up to 3 h in nulliparas without epidural analgesia, and up to 4 h with it, provided that labor progress is documented [10–12].

Extending the second stage increases vaginal delivery chances, but also raises obstetric risks, including postpartum hemorrhage (PPH), severe perineal lacerations, operative vaginal deliveries, and chorioamnionitis. Data regarding neonatal outcomes remain conflicting [13–21].

In a 2020 clinical opinion, Nelson et al.[22] questioned the safety of extending the second stage up to 4 h, noting that this guideline is based on consensus rather than evidence. Managing nulliparas with epidural analgesia is particularly challenging, since they face higher CD rates if not given adequate time for fetal head descent [23], yet also experience unfavorable vaginal delivery outcomes as the second stage lengthens [24].

While second-stage duration correlates with adverse obstetric outcomes, [13, 25] data regarding the impact of an additional hour in nulliparas with prolonged second stage and epidural are lacking.

A recent study [26] evaluated nulliparas with epidural analgesia and second-stage labor exceeding 3 h. They found that allowing a fourth hour increased vaginal delivery rates without harming mother or neonate, yet their cohort was relatively small.

This study aimed to evaluate obstetric outcomes in nulliparas with epidural analgesia who exceeded 4 h of second-stage labor, compared to those with 3–4 h, providing data to inform current practice. Our hypothesis was that patients with a second stage lasting more than 4 h would demonstrate higher rates of adverse obstetric outcomes, particularly those related to birth trauma, as the fetal head remains impacted within the maternal pelvis for a prolonged period.

## Methods

This study was a retrospective cohort conducted at a single tertiary medical center in Kfar Saba, Israel, between January 1, 2014, and January 31, 2021. Inclusion criteria were term, singleton deliveries of nulliparous patients with epidural analgesia who had a second stage duration of > 3 h.

Exclusion criteria included patients undergoing a CD during the first stage of labor and patients with a second stage duration of less than 3 h in order to evaluate only patients with prolonged second stage and to specifically evaluate the implication of extending the second stage duration an additional hour. Multiple gestations, pregnancies with known genetic/structural anomalies and medical records with missing obstetric outcomes were also excluded.

Patients with a second stage duration > 4 h (study group) were compared to patients with a second stage duration of 3–4 h (controls) in terms of maternal and labor characteristics, as well as obstetric and neonatal outcomes.

Monitoring was maintained throughout the labor process, including hourly assessment of maternal vital signs and continuous electronic fetal monitoring. All deliveries presented with satisfactory maternal and fetal conditions as well as documented advancement during the second stage—specifically, fetal head descent below the ischial spines—supporting the decision for conservative management and anticipation of vaginal delivery. In these cases, patients were counseled regarding the option to continue with labor for an additional hour. This included an explanation of the potential obstetric and neonatal risks associated with prolonged second stage. Only patients who understood the risks and chose to proceed were allowed this additional time.

According to our departmental protocol, all patients reaching one hour at the second stage undergo a full evaluation by a senior obstetrician, including a pelvic examination for fetal head station and position, as well as a sonographic evaluation of fetal head position. Oxytocin is administered if the station is at the level of ischial spines or above. Internal rotation is attempted if an occiput posterior/transverse position is present. Pushing is initiated after one hour in the second stage unless the patient’s urge to push occurs earlier. Pushing is guided by the attending midwife, taking into consideration the woman’s physical ability, comfort, and willingness to continue. It is not performed continuously but rather in intervals, with breaks as needed or requested by the patient to avoid exhaustion and support maternal well-being.

The primary outcome was a composite measure of neonatal birth trauma, defined as the occurrence of subgaleal hematoma, shoulder dystocia, or associated injuries (Erb’s palsy, clavicular fracture, and humeral fracture). The incidence of each component was also analyzed individually. Maternal birth trauma was assessed separately, defined as the occurrence of third- or fourth-degree perineal lacerations.

Secondary outcomes included mode of delivery and neonatal adverse outcomes not related to birth trauma.

### Data collection

Data were retrieved from the electronic databases of our obstetric triage, delivery room, and neonatal units. Information collected included:

1. *Maternal demographics*: age, BMI (kg/m.^2^), obesity (BMI > 30), smoking status, diabetes mellitus (pregestational or gestational) [27], and hypertensive disorders (chronic and/or gestational, defined according to the ACOG guidelines).[28]
2. *Delivery characteristics*: gestational age, labor induction, intrapartum fever (≥ 38 degree Celsius during delivery or up to 24 h from delivery), amniotic fluid color (clear, meconium stained or bloody), fetal head position and duration of second stage.
3. *Delivery outcomes*: mode of delivery (normal vaginal delivery [NVD], vacuum extraction [VE], or CD), rates of VE or CD preformed due to non-reassuring fetal heart rate (NRFHR), rates of failed VE, maternal blood loss (objectively calculated using a gravimetric machine) [29], rates of PPH [30], retained placenta, and third- or fourth-degree perineal lacerations.
4. *Neonatal outcomes*: 5-min Apgar score, umbilical cord pH, fetal sex, neonatal birthweight (small or large for gestational age (SGA, LGA), diagnosed according to local birthweight charts[31]), macrosomia (defined as BW ≥ 4000 g), NICU admission, shoulder dystocia, Erb’s palsy, clavicular fracture, humeral fracture, subgaleal hematoma, hypoglycemia (neonatal blood glucose < 40 mg/dL), sepsis, respiratory distress, anemia, need for blood transfusion, cerebral hemorrhage and hypoxic-ischemic-encephalopathy.

Neonatal diagnoses were determined by the senior pediatrician at delivery and during neonatal hospitalization, according to international standards, relevant blood samples and imaging (ultrasound and magnetic resonance imaging).

Shoulder dystocia was defined as a failure to deliver the fetal shoulder(s) after initial attempts using standard traction techniques, or when ancillary obstetrical maneuvers were required to achieve delivery of the shoulders.

### Ethics approval

The study was approved by the Medical Center Institutional Ethics Committee in September 2021, approval number 0167-21-MMC. Due to the retrospective nature of the data collection, the Ethics Committee exempted the authors from obtaining individual informed consent.

### Statistical analysis

Categorical variables were analyzed using Chi-square or Fisher’s exact tests, as appropriate, based on cell counts. Continuous variables were tested for normality using the Shapiro–Wilk test. Variables with a normal distribution were compared using independent-samples t-tests, while those with non-normal distributions were compared using Mann–Whitney U-tests.

To assess independent associations between second stage duration and the outcomes of interest (birth trauma and mode of delivery), multivariable logistic regression analyses were performed. This method was chosen to control for potential confounding factors and to determine which variables independently contributed to the outcomes. Potential confounders were both statistically different between groups in the univariate analysis and had clinical relevance supported by the literature. Results were reported as adjusted OR and 95% CI. P < 0.05 was considered significant. All analyses were performed using SPSS-28 (IBM Corp., Armonk, NY, USA).

### Sample size calculation

Power analysis was calculated for the most common outcome within the trauma outcomes measured: third- and fourth-degree perineal lacerations. Based on a previous report, rates of third- and fourth-degree perineal lacerations are 5.4% among nulliparas [32]. Assuming those rates will double with second stage prolongation to > 4 h, the sample size calculation indicated that at least 400 participants were needed for each arm of the study, using alfa of 0.05 and 80% power.

## Results

**Fig. 1:**
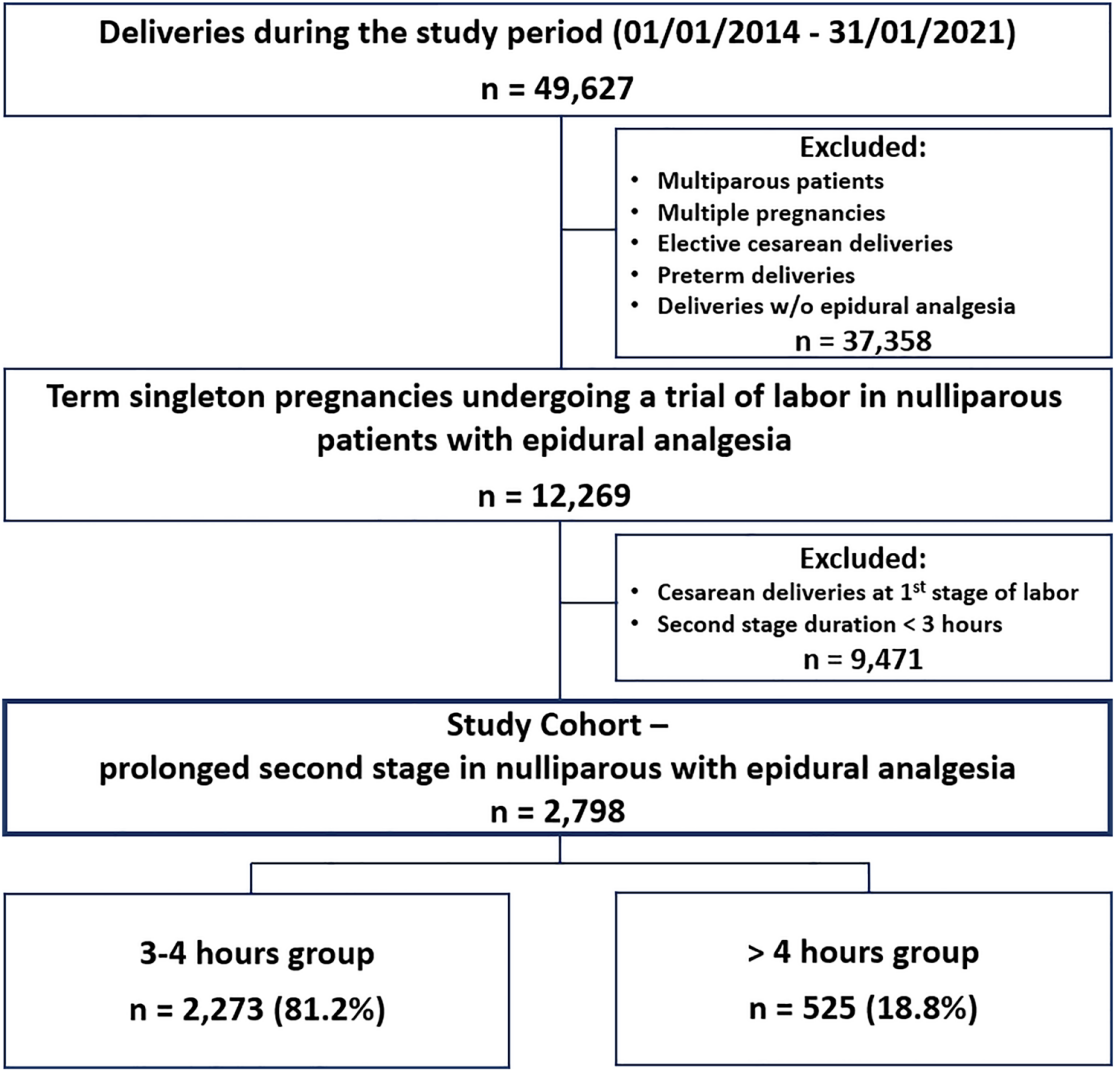
Flowchart describing the study population

During the study period, 49,627 patients delivered in our institution, of which 12,269 had a term, singleton first delivery with epidural analgesia. In total, 2798 met the inclusion criteria. Among them, 2273 (81.2%) had a second stage duration of 3–4 h (mean 3.42 ± 0.28 h, median 3.40, IQR 3.18–3.67 h) and 525 (18.8%) had a second stage duration of > 4 h (mean 4.38 ± 0.42 h, median 4.28, IQR 4.12–4.52 h) (Fig. 1).

### Maternal characteristics

The group with second stage > 4 h was characterized with higher rates of diabetes mellitus and hypertensive disorders compared to the controls (15.4% vs. 11.5%, p = 0.013 and 8.0% vs. 5.5%, p = 0.026, respectively). All other characteristics were similar (Table 1).

**Table 1:** Baseline characteristics of the study groups

Variable	Second stage > 4 h (n = 525)	Second stage 3–4 h (n = 2273)	p-value
Maternal age (years)	29.1 ± 4.9	28.8 ± 4.7	0.241
Gestational age at delivery (weeks)	39.5 ± 1.1	39.5 ± 1.1	0.694
Obesity (BMI > 30, kg/m^2^)	34 (10.2%)	125 (9.3%)	0.644
BMI, kg/m^2^	23.6 ± 5.1	23.3 ± 4.9	0.399
Smoking	24 (4.6%)	106 (4.7%)	0.928
DM	81 (15.4%)	261 (11.5%)	**0.013**
Hypertensive disorders	42 (8.0%)	124 (5.5%)	**0.026**

DM- includes pre-gestational and gestational diabetes mellitus; Hypertensive disorders—including chronic hypertension, gestational hypertension and preeclampsia

### Delivery characteristics

Labor characteristics were similar between the groups, including rates of labor induction, intrapartum fever, meconium stained amniotic fluid and occiput posterior fetal head position. Maternal blood loss during delivery was more excessive in the second stage > 4 h group compared to the controls (382 ± 230 ml vs. 345 ± 210 ml, p = 0.002); yet, rates of PPH, retained placenta, and third- or fourth-degree perineal lacerations did not differ between groups (Table 2).

**Table 2:** Labor and delivery characteristics

Variable	Second stage > 4 h (n = 525)	Second stage 3–4 h (n = 2273)	p-value
Induction of labor	201 (40.0%)	813 (36.7%)	0.171
Intrapartum fever	54 (10.3%)	204 (9.0%)	0.349
Meconium stained amniotic fluid	75 (14.8%)	363 (16.6%)	0.311
Position occiput posterior	60 (20.5%)	180 (18.9%)	0.524
*Mode of delivery *			
NVD	124 (23.6%)	1149 (50.5%)	** < 0.001**
VE	294 (56.0%)	963 (42.4%)	** < 0.001**
CD	103 (19.6%)	158 (7.0%)	** < 0.001**
VE due to NRFHR (within VE)	47 (16.0%)	484 (50.3%)	** < 0.001**
CD due to NRFHR (within CD)	8 (7.8%)	36 (22.8%)	**0.002**
Failed VE (within CD)	23 (22.3%)	22 (13.9%)	** < 0.001**
Second stage duration (hours)	4.38 ± 0.42	3.42 ± 0.28	** < 0.001**
Maternal blood loss (ml)	382 ± 230	345 ± 210	**0.002**
Post-partum hemorrhage	63 (12.0%)	235 (10.3%)	0.266
Retained placenta	20 (3.8%)	69 (3.0%)	0.362
Third- or fourth-degree perineal laceration	12 (2.3%)	60 (2.6%)	0.644

Continuous variables are presented as mean ± SD and categorical variables as n (%)

NVD-normal vaginal delivery; VE-vacuum extraction; CD-cesarean delivery; NRFHR-non-reassuring fetal heart rate

### Mode of delivery

Compared to the second stage 3–4 h group, the second stage > 4 h group had a lower rate of vaginal delivery (80.4% vs. 93.0%, p < 0.001), relatively higher rates of VE (56.0% vs. 42.4%, p < 0.001) and higher rates of CD (19.6% vs. 7.0%, p < 0.001). The 3–4 h group was characterized by higher rates of CD and VE due to non-reassuring fetal heart rate (22.8% vs. 7.8% for CD, p < 0.001 and 50.3% vs. 16.0% for VE, p < 0.001). Failed VE was almost twice as high in the > 4 h group compared to 3–4 h group (22.3% vs. 13.9%, p < 0.001) (Table 2).

### Neonatal outcomes

The second stage > 4 h group had higher rates of macrosomia and LGA birthweights (7.0% vs. 4.0%, p < 0.003 and 11.8 vs. 8.9%, p = 0.039, respectively). Shoulder dystocia and subgaleal hematoma were more common in the > 4 h group (2.7% vs. 1.1%, p < 0.001 and 9.0% vs. 3.3%, p < 0.001, respectively). The composite neonatal trauma outcome was higher in the > 4 h group (11.2% vs. 4.1%, p < 0.001). Neonatal Apgar scores, umbilical cord pH, NICU admission rates and composite neonatal outcome unrelated to birth trauma were similar between the groups (Table 3).

**Table 3:** Neonatal characteristics and outcomes in study group compared to control

Variable	Second stage > 4 h (n = 525)	Second stage 3–4 h (n = 2273)	p-value
Fetal sex, female	252 (48.0%)	1152 (50.7%)	0.268
Neonatal birth weight, (g) mean ± SD	3373 ± 403	3301 ± 403	** < 0.001**
SGA neonate	27 (5.1%)	165 (7.3%)	0.084
LGA neonate	62 (11.8%)	202 (8.9%)	**0.039**
Macrosomia	37 (7.0%)	91 (4.0%)	**0.003**
Apgar < 7 at 5 min	7 (1.3%)	21 (0.9%)	0.396
Cord pH < 7.1	15 (3.9%)	35 (2.6%)	0.155
Shoulder dystocia*	14 (2.7%)	24 (1.1%)	**0.004**
Subgaleal hematoma	47 (9.0%)	74 (3.3%)	** < 0.001**
NICU hospitalization	7 (1.3%)	31 (1.4%)	0.957
Composite neonatal outcome**	48 (11.5%)	166 (9.2%)	0.149
Composite neonatal trauma outcome***	59 (11.2%)	94 (4.1%)	** < 0.001**

*SD* standard deviation; *n* number; Data are presented as mean ± SD or number (rate), as appropriate

*SGA* small for gestational age - birth weight <10th percentile, *LGA* large for gestational age - birthweight >90th percentile

*Shoulder dystocia includes its related injuries: Erb’s palsy, clavicular fracture, and humeral fracture

**Composite neonatal outcome includes one or more of the following: hypoglycemia, sepsis, respiratory distress, anemia/blood transfusion, cerebral hemorrhage, hypoxic-ischemic-encephalopathy:

***Composite neonatal trauma outcome includes one or more of the following: subgaleal hematoma, shoulder dystocia and its related injuries: Erb’s palsy, clavicular fracture, and humeral fracture

### Multivariable logistic regression analysis

**Fig. 2:**
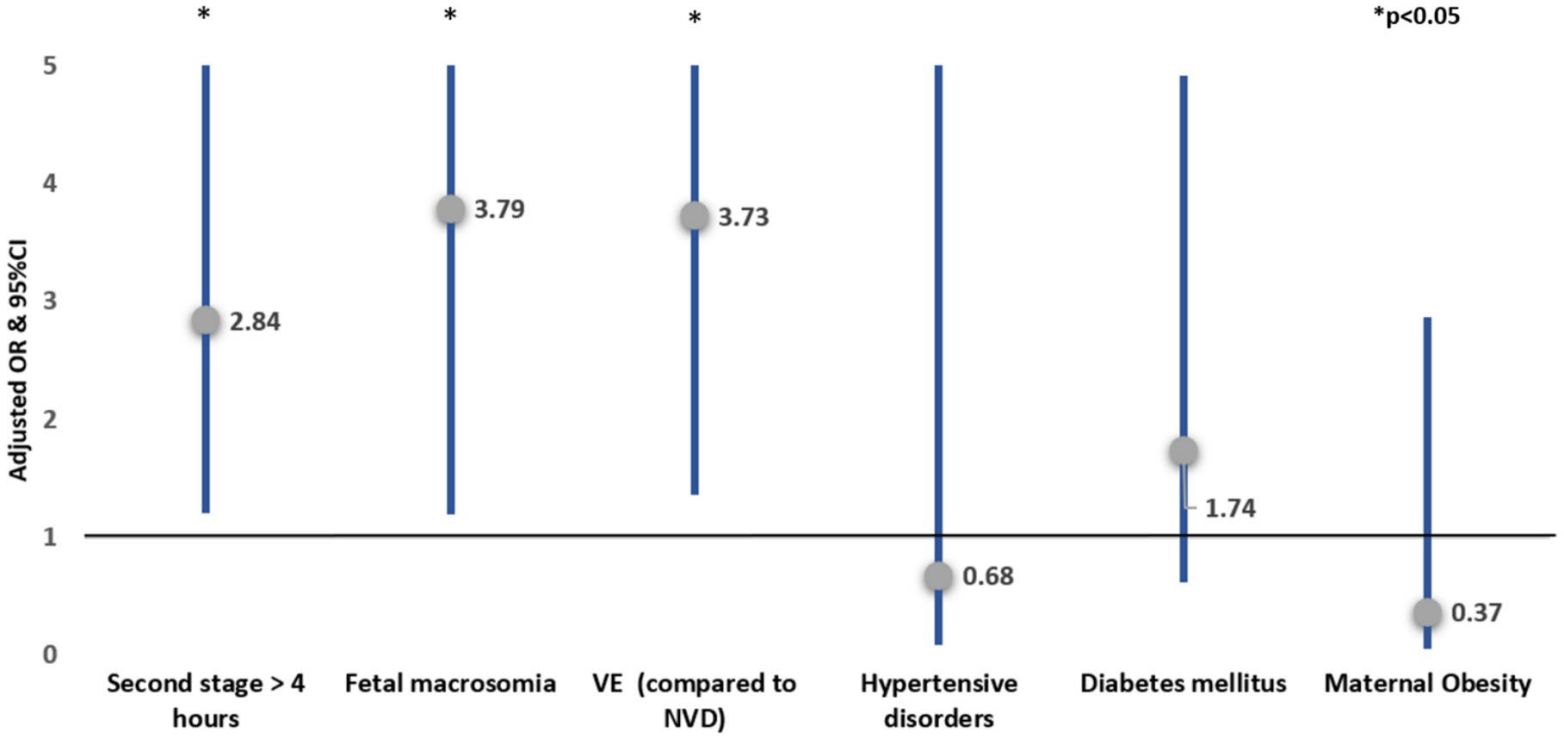
Multivariable logistic regression analysis of the risks of shoulder dystocia

**Fig. 3:**
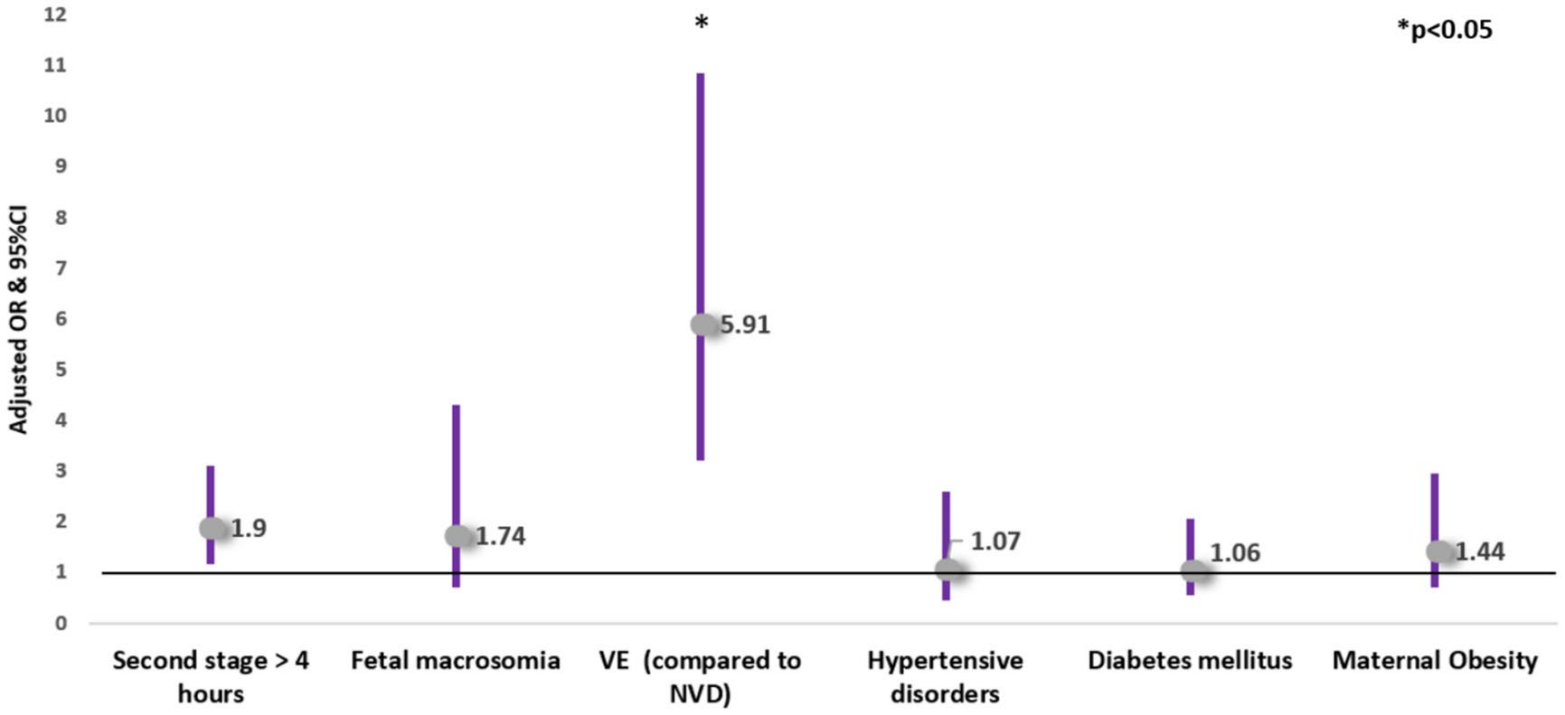
Multivariable logistic regression analysis of the risks of subgaleal hematoma

**Fig. 4:**
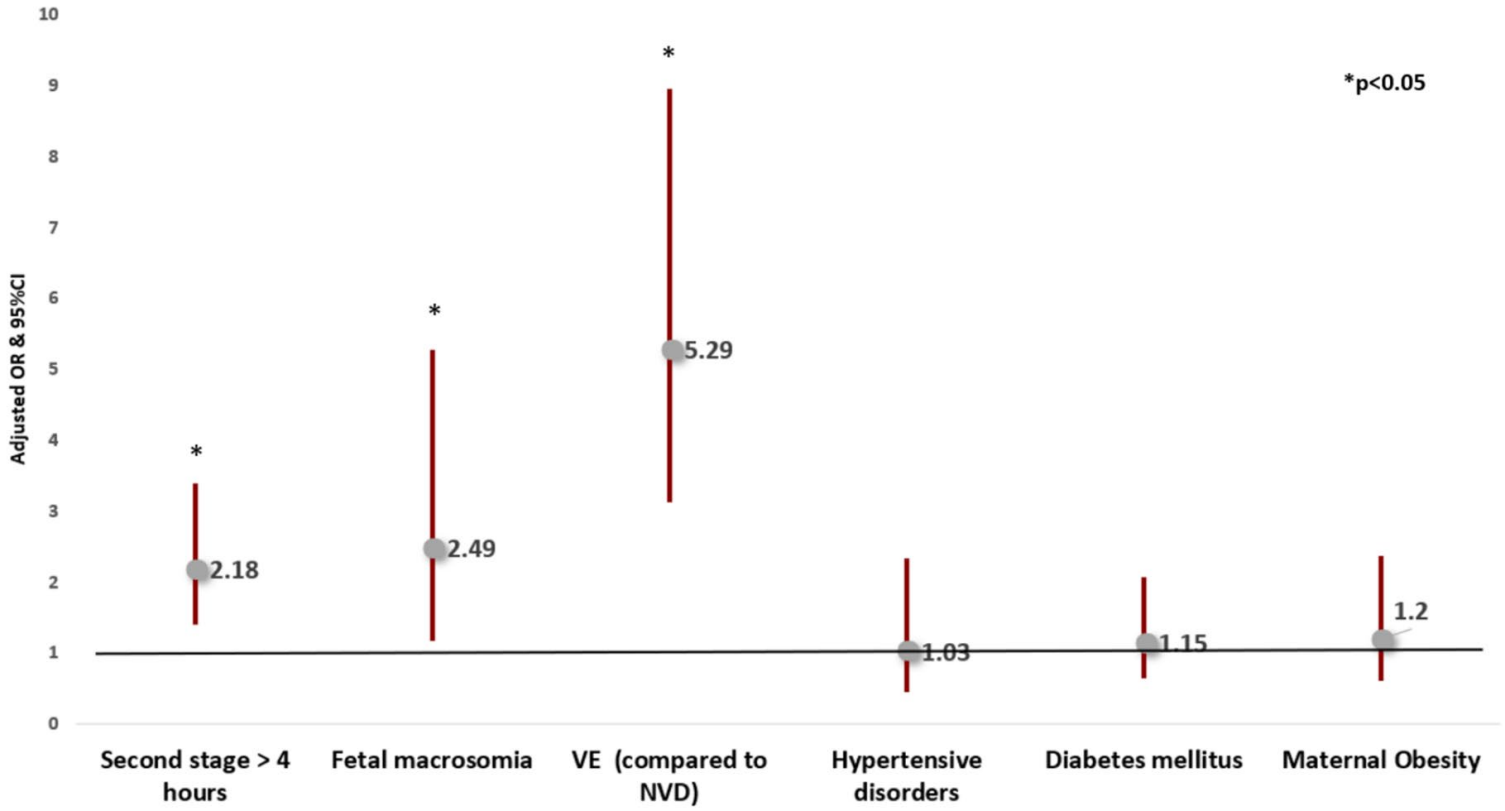
Multivariable logistic regression analysis of the risks of composite neonatal trauma outcome

**Fig. 5:**
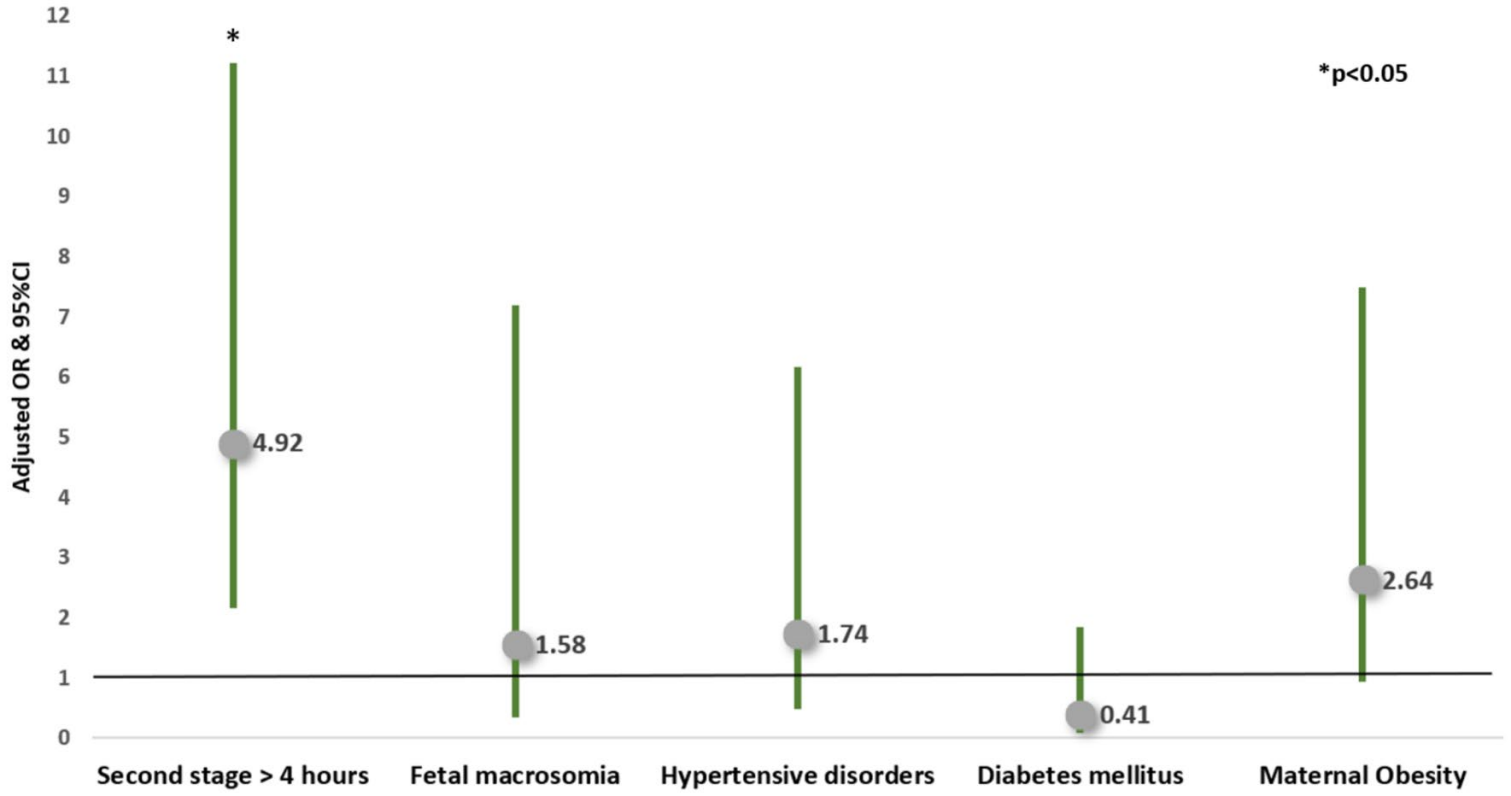
Multivariable logistic regression analysis of the risks of failed vacuum extraction

Applying multivariable logistic regression adjusted for confounders, second stage duration > 4 h (compared to 3–4 h) was *independently* associated with increased risk of shoulder dystocia (aOR 2.84, 95% CI 1.21–6.67), with increased risk of subgaleal hematoma (aOR 1.90, CI 1.16–3.12) and with an increased risk of failed VE, (aOR 4.92, CI 2.15–11.22). Second stage duration > 4 h was also associated with an increased risk of the composite neonatal trauma outcome (aOR 2.18, 95% CI 1.40–3.39) (Table 4, Figs. 2, 3, 4 and 5).

**Table 4:** Multivariable logistic regression analyses—the risks of shoulder dystocia, subgaleal hematoma, the composite neonatal trauma outcome and failed vacuum extraction

**Variable**	**aOR**	**95% CI**	**P-value**
*Risk of shoulder dystocia*			
*Second stage* > *4 h*	***2.84***	***1.21–6.67***	***0.017***
Fetal macrosomia	**3.79**	**1.20–12.01**	**0.024**
VE (compared to NVD)	**3.73**	**1.36–10.23**	**0.010**
Hypertensive disorders	0.68	0.09—5.27	0.715
Diabetes mellitus	1.74	0.62–4.92	0.294
Maternal obesity	0.37	0.05–2.87	0.342
*Risk of Subgaleal Hematoma*			
*Second stage* > *4 h*	***1.90***	***1.16—3.12***	***0.011***
Fetal macrosomia	1.74	0.70–4.32	0.236
Vacuum extraction (compared to NVD)	**5.91**	**3.22–10.84**	** < 0.001**
Hypertensive disorders	1.07	0.44–2.59	0.882
Diabetes mellitus	1.06	0.54–2.05	0.874
Maternal obesity	1.44	0.70–2.95	0.321
*Risk of the composite neonatal trauma outcome*			
*Second stage* > *4 h*	***2.18***	***1.40–3.39***	** < *0.001***
Fetal macrosomia	**2.49**	**1.18–5.28**	**0.017**
Vacuum extraction (compared to NVD)	**5.29**	**3.13–8.95**	** < 0.001**
Hypertensive disorders	1.03	0.45–2.34	0.953
Diabetes mellitus	1.15	0.64–2.07	0.649
Maternal obesity	1.20	0.61–2.38	0.603
*Risk of failed vacuum extraction*			
*Second stage* > *4 h*	***4.92***	***2.15–11.22***	** < *0.001***
Fetal macrosomia	1.58	0.35–7.20	0.551
Hypertensive disorders	1.74	0.49–6.17	0.394
Diabetes mellitus	0.41	0.09–1.85	0.248
Maternal obesity	2.64	0.94–7.48	0.067

*aOR* adjusted odds ratio, *CI* confidence interval, *NVD* normal vaginal delivery, *VE* vacuum extraction

Multivariable regression model was adjusted for confounders that were significantly different between groups in the univariate analysis, and were thought to affect the outcomes evaluated. These factors included maternal obesity, hypertensive disorders, diabetes mellitus, fetal macrosomia and mode of delivery. A total of 1660 cases were included in the model. Results are reported as adjusted odds ratios and 95% confidence intervals. VE, Vacuum extraction. NVD, Normal Vaginal Delivery**.**

Multivariable regression model was adjusted for confounders that were significantly different between groups in the univariate analysis, and were thought to affect the outcomes evaluated. These factors included maternal obesity, hypertensive disorders, diabetes mellitus, fetal macrosomia and mode of delivery. A total of 1660 cases were included in the model. Results are reported as adjusted odds ratios and 95% confidence intervals. VE, Vacuum extraction. NVD, Normal Vaginal Delivery.

Multivariable regression model was adjusted for confounders that were found significantly different between groups in the univariate analysis, and were thought to affect the outcomes evaluated, including maternal obesity, hypertensive disorders, diabetes mellitus, fetal macrosomia and mode of delivery. A total of 1660 cases were included in the model. Results are reported as adjusted odds ratios and 95% confidence intervals. VE, Vacuum extraction. NVD, Normal Vaginal Delivery.

Multivariable regression model was adjusted for confounders that were significantly different between groups in the univariate analysis, and were thought to affect the outcomes evaluated, including maternal obesity, hypertensive disorders, diabetes mellitus, fetal macrosomia and mode of delivery. A total of 1660 cases were included in the model. Results are reported as adjusted odds ratios and 95% confidence intervals.

## Discussion

This study demonstrates that in nulliparous patients receiving epidural analgesia, a prolonged second stage of labor exceeding 4 h is significantly associated with an increased risk of the composite neonatal trauma outcome, as well as with shoulder dystocia, subgaleal hematoma, and failed VE. A second stage duration > 4 h, compared to 3–4 h, resulted in lower rates of normal vaginal deliveries and relatively higher rates of VE and CD, especially due to arrested labor.

### Novelty of the current study

This study provides the largest and most detailed cohort analysis to date, specifically addressing the impact of extending the second stage of labor beyond 4 h in nulliparous patients receiving epidural analgesia. Unlike previous studies that either pooled nulliparas and multiparas or lacked stratification beyond the 3 h threshold, our analysis focuses exclusively on patients already meeting the definition of a prolonged second stage and examines the incremental risks of extending it further. This is a critical gap in the literature, as current guidelines permit prolongation beyond 3 h [8], yet robust evidence on maternal and neonatal outcomes beyond 4 h has been limited. The only prior study addressing this question, by Collinot et al. [26], reported no increase in morbidity, but their small sample size (n = 111) may have lacked power to detect rare but serious outcomes. By providing detailed, indication-specific data, our findings offer new insights that are directly relevant to clinical decision-making in the management of prolonged second stage labor.

## Results

We found that extension of the second stage duration to > 4 h allowed most patients to achieve a vaginal delivery (with 80.4% of the patients having either a NVD or VE). These results are in agreement with a previous randomized controlled trial reporting approximately 80% success in vaginal deliveries with prolongation of the second stage to > 3 h compared to 2–3 h [33]. Of note, Rosenbloom et al. [34] found that primary CD rates were not reduced after implementing the new ACOG guidelines [8] with the extension to > 4 h.

However, evaluation of the safety in this second stage prolongation in our cohort, revealed concerning results. We observed higher rates of the composite neonatal trauma outcome, shoulder dystocia, subgaleal hematomas and failed VE in patients with second stage duration of > 4 h, as compared to 3–4 h, as well as increased maternal intrapartum blood loss. Although Collinot et al. [26] reported no increase in maternal or neonatal morbidity with prolonging the second stage of labor to 4 h, their relatively small cohort perhaps limits these findings. Zipori et al. [15] evaluated nulli- and multiparas with and without epidural and in agreement with our findings, reported higher rates of shoulder dystocia and third- or fourth-degree perineal lacerations and after implementation of the ACOG guidelines, as well as higher rates of cord pH < 7.0 and NICU admissions. Grantz et al. [35] demonstrated that among nulliparas with epidural analgesia, prolonging the second stage from 3 to 4 h increased the rates of vaginal deliveries accompanied by a composite maternal or neonatal morbidity up to 7.6%.

It should be noted that although the prevalence of birth trauma tends to be higher during VE compared to NVD [36, 37], it is more prevalent with increased duration of the second stage, even when comparing VE alone. Previous studies demonstrated higher rates of birth trauma during VE when performed in the presence of prolonged second stage [24, 38]. Our multivariable regression analyses suggest an *independent association* with shoulder dystocia and subgaleal hematoma, in the presence of second stage of labor > 4 h. Hence, this association cannot be fully attributed to the increase in VE rates observed in the > 4 h group.

Obstetric morbidity becomes even more substantial in the face of a failed VE. We found excessive rates of failed VE in the > 4 h group compared to controls (22.3% vs. 13.9%, p < 0.001). This finding is novel and rather concerning, considering that failed VE is associated with significant maternal and neonatal adverse outcomes and even perinatal death, when compared to both a successful VE and a second-stage CD without a prior attempted VE [39, 40]. Failed VE also carries long-term risks, including higher rates of preterm delivery in subsequent pregnancy [41]. These implications should be thoroughly discussed before attempting to achieve a vaginal delivery at any cost.

It should also be noted that CDs performed after > 4 h in the second stage are much more complex compared to CDs performed after 1–3 h, including higher rates of uterine extensions and prolonged procedure duration [25]. Consequently, extending the duration of the second stage should be carefully evaluated.

We found higher rates of diabetes mellitus, LGA, and macrosomic neonates in the > 4-h group. These findings line with previous reports and reflect the association between diabetes and the risk of large birthweights and prolonged second stage [42–44]. We also observed higher rates of hypertensive disorders in this group, consistent with a study reporting longer second-stage in the presence of epidural analgesia among patients with gestational hypertension or superimposed preeclampsia [45]; however, data are inconclusive [46].

Individualized management should focus on balancing the benefits of prolonged labor with the risks associated with it. Our study revealed increased vaginal delivery rates with the second stage prolonged beyond 4 h. However, these deliveries were accompanied by an increased risk of birth trauma. These findings should be carefully weighed when assessing the safety and cost-effectiveness of prolonging the second stage in nulliparas with epidural [8, 10, 11], and should be clearly communicated to patients as part of a shared decision-making process. In patients with additional risk factors that do not favor a vaginal delivery, prolonging the second stage may not be advisable, and an earlier operative decision should be considered. For low-risk patients, however, shared decision-making should guide the approach. If, after being fully informed of the potential risks, the patient opts to continue labor for an additional hour, the option of attempting a VE should be thoroughly deliberated prior to the procedure, given that the risk of failure exceeds 20%.

The strengths of this study include the large cohort and detailed documentation, allowing us to thoroughly evaluate the second-stage duration among nulliparas with epidural analgesia. Data were retrieved from a single institution with similar medical protocols, creating a relatively homogenous cohort. The findings demonstrating an association between second-stage duration > 4 h and birth trauma are novel, and detail the possible risks associated with vaginal deliveries under these circumstances. Notably, this study was powered to rule out an increase in rates of third- or fourth-degree perineal lacerations in the > 4 h group, meaning this particular outcome related to birth trauma was not one of the dangers associated with extending the duration of the second stage.

The study had some limitations, including its retrospective design, that resulted in a lack of some information, including long term obstetric outcomes. Therefore, these outcomes could not be evaluated. Our department follows a strict protocol under which pushing is attempted after one hour in the second stage; thus, we could not evaluate the role of immediate versus delayed pushing and its implications on mode of delivery and birth trauma [17]. However, this allowed us to evaluate the role of second-stage duration without the additional confounding factors (oxytocin administration duration, the timing of pushing, etc.).

## Conclusions

Most nulliparas with epidural analgesia ultimately achieve vaginal delivery even after more than four hours in the second stage of labor. However, these deliveries are associated with increased risks of neonatal birth trauma and failed VE**.** These risks must be clearly communicated to patients through a shared decision-making process. Importantly, the decision to prolong the second stage should be individualized, with extended efforts reserved for patients without additional risk factors. In women at low risk of complications, a carefully monitored trial of continued labor may be reasonable. Further research is needed to refine existing guidelines and support evidence-based practice.

## Data Availability

Data will be made available from the corresponding author upon reasonable request.

## Declarations

### Generative AI in scientific writing

This study did not use generative AI during scientific writing.
